# Approaches for Ending Ineffective Programs: Strategies From State Public Health Practitioners

**DOI:** 10.3389/fpubh.2021.727005

**Published:** 2021-08-20

**Authors:** Emily Rodriguez Weno, Peg Allen, Stephanie Mazzucca, Louise Farah Saliba, Margaret Padek, Sarah Moreland-Russell, Ross C. Brownson

**Affiliations:** ^1^Prevention Research Center, Brown School, Washington University in St. Louis, St. Louis, MO, United States; ^2^Department of Surgery (Division of Public Health Sciences), Alvin J. Siteman Cancer Center, Washington University School of Medicine, Washington University in St. Louis, St. Louis, MO, United States

**Keywords:** termination, prevention and control, evidence-based practice, public health practice, implementation science

## Abstract

**Background:** Public health agencies are increasingly concerned with ensuring they are maximizing limited resources by delivering evidence-based programs to enhance population-level chronic disease outcomes. Yet, there is little guidance on how to end ineffective programs that continue in communities. The purpose of this analysis is to identify what strategies public health practitioners perceive to be effective in de-implementing, or reducing the use of, ineffective programs.

**Methods:** From March to July 2019, eight states were selected to participate in qualitative interviews from our previous national survey of US state health department (SHD) chronic disease practitioners on program decision making. This analysis examined responses to a question about “…advice for others who want to end an ineffective program.” Forty-five SHD employees were interviewed *via* phone. Interviews were audio-recorded, and the conversations were transcribed verbatim. All transcripts were consensus coded, and themes were identified and summarized.

**Results:** Participants were program managers or section directors who had on average worked 11 years at their agency and 15 years in public health. SHD employees provided several strategies they perceived as effective for de-implementation. The major themes were: (1) collect and rely on evaluation data; (2) consider if any of the programs can be saved; (3) transparently communicate and discuss program adjustments; (4) be tactful and respectful of partner relationships; (5) communicate in a way that is meaningful to your audience.

**Conclusions:** This analysis provides insight into how experienced SHD practitioners recommend ending ineffective programs which may be useful for others working at public health agencies. As de-implementation research is limited in public health settings, this work provides a guiding point for future researchers to systematically assess these strategies and their effects on public health programming.

## Introduction

Public health agencies are increasingly concerned with ensuring they are maximizing limited resources by delivering effective evidence-based programs to enhance population-level chronic disease outcomes (1–3). Despite the focus on use of effective interventions, ineffective programs too often continue at public health agencies. Our recent national survey of 643 state health department practitioners found, in a close-ended multiple choice question, that 50% of respondents indicated that programs sometimes or more often continue when they should have ended (4). However, there is little guidance for de-implementation, the process of abandoning an intervention that does not demonstrate efficacy or may cause harm, in public health settings (5, 6).

It is important to better understand de-implementation in public health settings for several reasons. First, public health departments have increasingly limited resources. An analysis of federal, state, and local public health budgets has shown that public health spending has fallen or remained flat over the last several years (7, 8). Making the best use of limited resources includes ending programs that are not effective or provide low value for the resources used. Second, public health agencies have an ethical obligation to be efficient with resources (1). As public health agencies are predominantly publicly funded and serve the public, often the most vulnerable, public health agencies need to get the most value from their interventions in order to serve most effectively as many people as possible (1, 7). De-implementation of interventions that are ineffective and replacing them with effective interventions is one way that public health agencies can meet their obligation of efficiency.

Harris et al. offer a framework for evidence-driven decision-making to guide disinvestment of ineffective healthcare practices and ensure effective resource allocation in healthcare (9–11). The framework starts with the principles of evidence-based practice that emphasize use of evidence from research and program evaluation findings along with staff and stakeholder input (12). The framework by Harris et al. (9) posits that organizations' funding, leadership, priorities, organizational readiness for change, staff expertise, and stakeholder engagement influence evidence-based decision making and resulting resource allocation. The authors propose that an organization's procedures and processes, ability to identify ineffective practices, support service capacity (e.g., to communicate research evidence), availability of local data to inform decision-makers, and program evaluation have bidirectional influences on each other that ultimately determine whether and how ineffective practices are disinvested and resources allocated more effectively (9, 13).

Additionally, researchers studying de-implementation in healthcare can learn from conceptualizations of the policy termination process developed by political scientists and applied to resource allocation considerations in a number of fields (14–17). Policy termination is defined as the planned conclusion or stoppage of specific government functions, organizations, policies, or programs (16). A government function is defined as a service provided by the government to its residents (15). Here external influences of termination proponents and opponents are emphasized. Those favoring termination of a governmental service, program, or policy may view it as an ineffective approach, less important than another government function that should receive the resources instead, or an impediment to adoption and implementation of a more suitable approach (14). Postulated barriers to policy termination include initial policy complexity or design for longevity, avoidance of politically costly conflicts over termination, political ideologies and other aspects of the political environment, institutional resistance, and financial and legal costs (14–17).

Despite these works on de-investment in healthcare and policy termination in government, there remains a gap in the literature focused on de-implementation in public health settings (5, 18–22). Much of the current work on de-implementation in health-related organizations have been conducted in healthcare settings (22, 23). Public health agencies are distinctly different than healthcare settings as they typically have different funding mechanisms, stakeholders, and more of a population focus (6, 24). These differences likely lead to unique factors affecting de-implementation in public health settings. A notable exception is a work by McKay et al. that analyzed existing de-implementation frameworks and theory to develop a conceptual narrative on the de-implementation process in public health settings (5). McKay et al. recognized the unique and complex process of de-implementation in public health agencies and the lack of existing research on the topic. The authors call for more evidence and research on factors and strategies that encourage successful de-implementation of ineffective programs in public health settings to better guide public health practitioners.

The purpose of this analysis is to identify what strategies public health practitioners perceive to be effective in de-implementing, or reducing the use of, ineffective programs.

## Methods and Materials

In Spring 2019, we selected eight states to participate in key informant interviews who previously participated in our national survey of state health department (SHD) chronic disease practitioners on program decision making. This analysis examined responses to the question: “What advice do you have for others who want to end an ineffective program.” Interviews were audio-recorded, and the conversations were transcribed verbatim through a transcription service, Rev.com (25). All transcripts were consensus coded, and themes were identified and summarized. Ethical approval for this study was granted by the Washington University in St Louis Institutional Review Board (IRB# 201812062).

### Study Participants and Recruitment

We selected participant states for geographic and population size representation and based on their self-reported frequency of mis-implementation from our 2018 national survey (4). We selected SHDs from each of the four US Census Bureau regions (Northeast, Midwest, South, and West). Mis-implementation in public health practice is the inappropriate continuation of ineffective interventions and de-implementation of effective interventions (18). States were selected to include both those that reported high and low mis-implementation frequency. We selected 4 SHDs whose chronic disease employees reported perceived frequency of mis-implementation in the highest quartile and 4 SHDs with reported perceived frequency of mis-implementation in the lowest quartile. After the SHDs were selected, as a courtesy, the team first contacted each state's chronic disease director *via* email to inform them of the study and that the study team would be contacting their programmatic staff. This communication was also used to invite the chronic disease director to participate and ask for any staff whose participation would be beneficial. One of the SHD chronic disease directors requested that we not contact their employees and the study team selected an alternative state to recruit from instead from the same quartile of perceived mis-implementation frequency.

After chronic disease directors were informed of the study, we reviewed position responses from the initial national survey to invite SHD chronic disease employees with position types likely to be involved in and have broad knowledge of how decisions were made about continuing and ending programs. We first emailed SHD employees from selected SHDs who responded to our previous national survey. Snowball sampling was then used as we asked each interview participant to recommend others at their SHD who would be a good fit for the interviews. When this did not yield a sufficient number of participants per state, we used the National Association of Chronic Disease Directors (NACDD) membership list to select additional potential participants from the selected SHDs. We contacted over 200 individuals to participate in our interviews; however, as anticipated, some did not respond to our repeated emails and attempts to contact by phone. Those who declined cited lack of time or inadequate knowledge on decision-making. We contacted potential participants to give them the opportunity to consent to schedule an interview or decline up to three times *via* email and two times *via* phone. Participants were offered a $40 amazon gift card or a $40 donation to a public health charity of their choice.

### Interview Guide Development

The interview guide focused on organizational, individual, and external factors that relate to decision-making about programs in SHDs. Specifically, the questions in this interview guide sought to elicit more about decision-making processes related to ineffective programs that continue in SHDs. We omitted the other type of mis-implementation, de-implementation of effective programs, because our 2018 national survey found that nearly 90% of SHD practitioner respondents believed the overwhelming reason for this type of mis-implementation was funding, of which SHD practitioners have limited control over (4). The interview guide had a description of the purpose of our research and included broad, open-ended questions followed by more specific questions to elicit detailed responses from participants. The questions were developed to better understand factors related to mis-implementation identified in our national survey (4). Questions asked for examples of ineffective programs that were continued, why and how decisions were made in SHDs to continue ineffective programs and about the influence of individual staff, organizational capacity, and external funding and policy environments on these decisions. We did not provide a definition of ineffective programs and instead allowed participants to self-define. We pilot tested the interview guide with a study advisor who was a recently retired SHD practitioner and made phrasing edits to questions based on their feedback. This analysis examined responses to one interview guide question: “What advice do you have for others who want to end an ineffective program?” We provided the interview guide questions to participants prior to the interview.

### Data Analysis

We used a deductive approach for our thematic analysis in that we developed a codebook to guide the process. The codebook was developed from the interview guide and included nine parent nodes with several sub-nodes. For this analysis, all responses to the question about “…advice for others who want to end an ineffective program” were coded into one node. Interviewers or another team member checked transcripts against the recordings and de-identified the transcripts. We coded and analyzed transcripts in NVivo 12 (26). We randomly assigned and distributed team members for coding. Two team members independently coded and then met to reach consensus on any discrepancies. Saturation was identified during this process when all codes and sub-codes had a variety of data and few new concepts emerged from new interviews at which point recruitment of new interview participants was ended. After the completion of consensus coding, team members identified and summarized sub-themes. For the current analyses, we focused on the thematic analysis of the node about “…advice for others who want to end an ineffective program.”

## Results

We completed interviews with 45 state health department practitioners (Table 1) from eight states. The average interview took 43 min (range 20–68 min). Most participants were at the program manager (64%) or section director level (22%), and all but one participant was female. Participants reported working at their agency for an average of 11 years and in public health for an average of 15 years. During the interviews participants demonstrated knowledge of and involvement in programmatic decision-making within their SHD. The following themes were identified from participants' responses to the interview question about advice for ending ineffective programs (Table 2): *(1) collect and rely on evaluation data; (2) consider if any of the program can be saved; (3) transparently communicate and discuss program adjustments (4) be tactful and respectful of partner relationships; (5) communicate in a way that is meaningful to your audience*.

**Table 1:** Demographic characteristics of state health department practitioners who participated in interviews on advice for ending ineffective programs, United States, 2019.

**Characteristic**	**Participants (*N* = 45) *n* (%)^a^**
**Gender**	
Female	44 (98)
Male	1 (2)
**Position**	
Program manager or coordinator	29 (64)
Director overseeing multiple programs in a section, bureau or division	10 (22)
Evaluator	2 (4)
Epidemiologist	2 (4)
Other (analyst, clinical care liason)	2 (4)
**Time spent in current position (years)**	
≤5	26 (58)
6–10	9 (20)
≥11	7 (16)
**Time spent in current agency (years)**	
≤5	17 (38)
6–10	10 (22)
≥11	17 (38)
**Time spent in public health overall (years)**	
≤5	4 (9)
6–10	13 (29)
≥11	26 (58)

a *Particiapnts came from 8 states representing all US Census Bureau regions including Northeast (3 states), South (2 states), Midwest (2 states), and West (1 state)*.

**Table 2:** Themes and sub-themes of approaches for ending ineffective programs, United States, 2019.

**Theme**	**Sub-theme**
1. Collect and rely on evaluation data	Have an evaluation process in place from the beginning
	Collect quantitative and qualitative data
	Use data to justify why the program needs to end
2. Consider if any of the program can be saved	Make sure it really needs to end
	Consider the ripple effects of ending the program
	Consider if adaptations or alternatives are possible
3. Transparently communicate and discuss program adjustments	Document and keep track of attempts to make the program work
	Communicate and be transparent about programs from the beginning
	Listen and be open-minded
4. Be tactful and respectful of partner relationships	Prevent burning bridges with partners
	Let partners know you value them
	Find ways to support partners who do good work
5. Communicate in a way that is meaningful to your audience	Tell stories
	Use written and concise communication
	Present relevant evaluation findings in an understandable way

### Collect and Rely on Evaluation Data

Participants frequently discussed how having evaluation in place helps end ineffective programs. Three major sub-themes emerged from this theme that included having an evaluation process in place from the beginning of a program, making sure to collect quantitative and qualitative data, and using data to justify why the program needs to end (Table 2). The most commonly cited reasons for eventually de-implementing a program were that evaluation findings showed the program was either not reaching the priority target population or was not having the desired impact, that funding or agency capacity was lacking, and lack of partner support or political will.

#### Sub-theme 1: Have an Evaluation Process in Place From the Beginning

Participants discussed how evaluation needs to be in place at the beginning of the program, including the importance of having staff or contractors skilled in evaluation. Examples of interview responses include the following:

“*Have an evaluation process along with the program planning process because if we do the implementation of the program and then expect an evaluation, that will never tell you where exactly that program was ineffective.” [Participant 1]*

“*Set evaluation parameters upfront, to really do your due diligence and do formative evaluation too to know what are contextual factors in the target populations or community in which you plan to serve through this program.” [Participant 2]*

#### Sub-theme 2: Collect Quantitative and Qualitative Data

Many participants highlighted not only collecting quantitative measures but also the importance of qualitative evaluation and anecdotal stories about how the program is working.

“*Make sure you have your evaluation data and both quantitative and qualitative evaluation data.” [Participant 3]*

“*Looking at the data, maybe gathering some anecdotal stories or qualitative data, but being really mindful of just looking at the outcomes before you make decisions.” [Participant 4]*

#### Sub-theme 3: Use Data to Justify Why the Program Needs to End

Participants also highlighted how valuable and important having data from evaluation is when you are making the case for ending an ineffective program. Participants stated that evaluation data can be used to convince decision-makers that are higher up in the SHD and other stakeholders, who may have dissenting opinions of program effectiveness, that a program is ineffective and needs to end.

“*Data. Show them the data. Save the data, use the data. If it's ineffective, you should be able to show that it's not working… lay out a logical argument that shows the money is being spent and the results aren't there.” [Participant 5]*

“*Those impacted… may not like it, and they may not necessarily 100% agree with the decision, but when you have the data and you have the evidence to support the decision and you're open and honest about that, I think it makes the blow a little bit easier.” [Participant 6]*

### Consider If Any of the Program Can Be Saved

A second theme that arose from our interviews was to think carefully about whether a program must end or if instead it could be adapted. Participants advised others to thoroughly consider what effects ending the program will have throughout the SHD. Three major sub-themes emerged within this theme which were making sure that a program really needs to end, considering the ripple effects of ending the program, and considering if adaptations or alternatives are possible.

#### Sub-theme 1: Make Sure It Really Needs to End

Participants acknowledged that ending a program is often difficult and advised others wanting to end an ineffective program to take a hard look and confirm the program must end. They highlighted that others should thoroughly examine program inputs and see if the program can bring about the desired outcomes.

“*Review all the inputs that are necessary to get the desired outputs are… if you go through that whole process and you see that there's nothing else that you can put in for inputs or resources you have or now you don't have available to you, if you're having to fudge it to make it just exist, but it's not going to be successful the way that it was planned to be, probably want to consider ending it.” [Participant 7]*

“*Before ending it completely really looking at all of the data and do an evaluation of the program first to make sure that it's really ineffective, and also I mean definitely talking with stakeholders.” [Participant 8]*

“*Be sure that the evidence is very clear as to why it's ineffective and to be sure that it's well documented and communicated.” [Participant 9]*

#### Sub-theme 2: Consider the Ripple Effects of Ending the Program

Another sub-theme that arose was participants' advice to thoroughly consider the effects of ending a program. Interview participants stated that practitioners need to consider how the ripple effects of ending a program, even an ineffective one, may create gaps in services that could affect other programs or stakeholders. It was advised that when working to end an ineffective program that practitioners develop a plan for how to address any undesirable effects that they identify.

“*[Consider] are there some ripple effects that can be seen based on the discontinuation of that program? … Even ineffective programs that can change your partnerships, they can affect inadvertently other programs.” [Participant 10]*

“*Make sure that you've considered all things in programs, most importantly who the program is going to impact, and you brought in sort of their thought process as well.” [Participant 11]*

“*Make sure you've talked to the stakeholders involved to assess what impacts would be felt and then mitigate that impact.” [Participant 3]*

#### Sub-theme 3: Consider If Adaptations or Alternatives Are Possible

A third identified sub-theme was to consider if adaptations or alternatives are possible to turn an ineffective program into an effective one. Participants advised that adapting programs where possible to make them effective can help to preserve programs and to prevent gaps in services. If a program really must end, participants advised practitioners to present alternatives to the ineffective program.

“*Decide whether the program really needs to end or if it is something that really just really need some minor tweaks.” [Participant 12]*

“*Find an effective alternative, or suggest an alternative as a solution to the problem that the ineffective solution was originally created for.” [Participant 13]*

“*You've been thinking through if we redirect these resources, what could we do with it and what the benefit could be. It's also helpful strategy to have some thoughts and comments related to that.” [Participant 14]*

### Transparently Communicate and Discuss Program Adjustments

A major theme from participants was to transparently communicate about programs and their adjustments. This theme related to keeping decision-makers regularly informed about the status of a program, soliciting their feedback, and documenting changes made to programs in attempts to make them effective. Three major sub-themes emerged on how to facilitate transparent communication, they included documenting and keeping track of attempts to make the program work, communicating and being transparent about programs from the beginning, and to listen and be open-minded.

#### Sub-theme 1: Document and Keep Track of Attempts to Make the Program Work

Participants advised documenting all efforts throughout the life of the program to make it work in order to show decision-makers how all options have been exhausted to make the program effective. One participant described it as doing “forensics” of how a program got lost along the way.

“*Any time that we can document, ‘Here are the steps that we've taken to remedy the situation,’ that has always been very beneficial in garnering support from leadership to end a program.” [Participant 15]*

“*Really document why this is an ineffective program to you and for at least a good six months or more… there really needs to be a lot of work shown and documentation of why it didn't work. Going forward you can also learn from all that and try to tweak things to make it even better the next time around.” [Participant 16]*

#### Sub-theme 2: Communicate and Be Transparent About Programs From the Beginning

Another major piece of advice was communicating with stakeholders and other staff throughout the life of the program so that there were no major surprises when it became clear the program was ineffective or so all can help to adjust the program along the way to make it effective.

“*Always be transparent even from day one, long before you think it's gonna be ineffective. Stay in close communications and so there are no surprises…” [Participant 17]*

“*I think communication, communication, communication. Certainly it is to engage the staff, engage the evaluator… So, I think it's all just really good transparent communication” [Participant 18]*

#### Sub-theme 3: Listen and Be Open-Minded

Finally, participants often mentioned listening and being open-minded in their conversations about programs to get a better understanding of a program's objectives and also to get those on board with ending the program who may have a personal attachment to programs.

“*When we go into those types of meetings where a decision has to be made, it's important to have a fine line of being prepared and coming with the data, but also being humble and open-minded that this program or this approach might be near and dear to someone's heart.” [Participant 6]*

*I would say to listen first. I listened for a while and then went to a few meetings and was going, “This just doesn't feel right to me.” Listen and understand what the goals and objectives are. Listening is first. Then ultimately, I did a bunch of research. [Participant 19]*

### Be Tactful and Respectful of Partner Relationships

Partnerships and their importance in ending ineffective programs were a major theme in our interviews. Participants highlighted how partners played a huge role in continuing or ending ineffective programs. It was recommended to be very tactful and respectful in how you work with partners when ending an ineffective program because often SHD practitioners want to work with partners again on other programs. Three major sub-themes emerged on how to be tactful and respectful of partner relationships, including to prevent burning bridges with partners, let partners know you value them, and finding ways to support partners who do good work.

#### Sub-theme 1: Prevent Burning Bridges With Partners

A major sub-theme in the ending of ineffective programs was to prevent burning bridges with partners that practitioners may want to work with again. Participants frequently spoke about speaking with partners about why a program needs to end in a very delicate way to preserve their working relationship. It was advised to give partners the full reasoning and rationale for the program ending to help them best understand.

“*Most individuals, in my experience, when you can show them the evidence and the data and show them what they're looking at, hear what they have to say, and if that's still the decision to discontinue at the end of the day, they are more likely to still come to the table on other opportunities that you may have… sometimes it has to be [program must end], but if it's not communicated well, you really can burn an opportunity or future partnerships.” [Participant 6]*

“*It may be that you never partner with them again, but I think, depending on how that ends, kind of signifies your relationship not only to that partner but to other partners that then may get worried about what the future holds for them.” [Participant 20]*

#### Sub-theme 2: Let Partners Know You Value Them

One way that participants suggested to preserve good relationships with partners was by letting them know their work and input are valued. Partners may be very invested in a program and it is key to remind good partners that their work is appreciated despite the fact the program needs to end.

“*You need to be very, very sensitive in how you communicate your rationale to the partners…It was very important to frame it in the way that, oh, you know, this program has helped many kids at the local level. All those partners that love the program so much, we need all of them to be on board with this shift as well.” [Participant 21]*

“*Find a way to manage the personal relationships, to make it clear that you value the other person's input, you value what they do, and you're invested in their success.” [Participant 13]*

#### Sub-theme 3: Find Ways to Support Partners Who Do Good Work

Another sub-theme was to preserve partner relationships when ending an ineffective program was to find ways to support partners that do good work even if the current program engagement with them must end. Participants suggested engaging good partners in other SHD programs or offering to support them in other ways.

“*Show how you can help them [partners] be successful even if that particular program is ending.” [Participant 13]*

“*There needs to be a way back in or a way of building and supporting some of our partners that typically do good work.” [Participant 20]*

### Communicate in a Way That Is Meaningful to Your Audience

The final major theme we identified in participants' advice was to communicate in a way that is meaningful to the audience. In order to best communicate why a program needs to end, participants advised both understanding the audience of the message and then deciding which way would be most useful to communicate with them. Three major sub-themes emerged on how to best communicate meaningfully which was to tell stories, use written and concise communication, and to present relevant findings in understandable ways.

#### Sub-theme 1: Tell Stories

Participants advised using stories to help convey why a program needs to end. They stated that using stories, with data, can be more memorable and persuasive than presenting data alone when convincing a decision maker that a program is ineffective and needs to end.

“*I've always taken away from listening to legislatures is the thing that sways them the most is the personal stories… personal stories really amplify that data.” [Participant 22]*

“*Speak up, use your data, tell your story.” [Participant 23]*

#### Sub-theme 2: Use Written and Concise Communication

Participants also spoke to the value of concise written communication for decision-makers who are often busy and don't have time to read extensive reports. They noted that concise one-pagers can be useful for this purpose. To develop this one-pager, participants advised “putting yourself in their [decision-makers'] shoes” to identify information that is pertinent to share with the decision-maker and what could be left out to honor their very busy schedules.

“*What's the best way to communicate that? Typically something written short. I like to say, you know, decision makers typically only read one page but we try to give them the whole thing. I would say something written and very concise.” [Participant 24]*

“*I'm a big proponent of a one-pager. I think as we go up the chain, people read less and less, so it might have to be a half-a-page, but any sort of document that concisely shows some background and then what sort of data's been collected, what sort of effort the state has made.” [Participant 15]*

#### Sub-theme 3: Present Relevant Evaluation Findings in an Understandable Way

Finally, participants highlighted the importance of not just collecting evaluation data but also disseminating it in a way that anyone could understand. They stated that the information from evaluations needs to be understandable by those who make decisions about programs and to avoid letting the data collected go to waste.

“*Present the results from your evaluations in a manner that is understandable by anyone… it used to be very common that it would have an evaluation that would just sit on a shelf and just because you were required by CDC to do that evaluation.” [Participant 25]*

“*So going into those conversations prepared, ‘What would I want to know or what would I want to see if I'm going to be told this information?”’ [Participant 6]*

## Discussion

Given the limited capacity and funds in public health agencies (12), it is crucial to efficiently manage resources to improve population-wide health. In doing so, one component of increasing the effectiveness and efficiency of programs involves ensuring that ineffective programs end. To better understand de-implementation strategies used in public health settings, this analysis examined advice from state health department practitioners on how to end ineffective programs. Using qualitative interviewing and thematic analysis, we identified five major themes in practitioner advice to end ineffective programs: (1) collect and rely on evaluation data; (2) consider if any of the program can be saved; (3) transparently communicate and discuss program adjustments; (4) be tactful and respectful of partner relationships; (5) communicate in a way that is meaningful to your audience.

Findings from the present study align with conceptualizations of the public policy termination process and the healthcare resource allocation model by Harris et al. (9). Disinvestment in a healthcare practice or termination of a government service, program, or policy is difficult, as acknowledged by the interview participants and by the models' authors. Harris et al. (9) built capacity to effectively communicate research evidence and local data to decision-makers into their model of evidence-based decision making for resource allocation. Interview participants discussed the importance of communication content and styles to increase stakeholder acceptability of the need for the program to end while maintaining positive relationships with partnering organizations and other stakeholders. They recommended not only explaining the research and local evaluation evidence, but also demonstrating that adaptations and alternatives had been explored. The political science theorists emphasize influences of external stakeholder groups in whether or not a government service, program, or policy is terminated (14, 15). While interview participants did not typically use political science terminology, they emphasized the importance of maintaining relationships with and respecting external stakeholders while considering and communication about de-implementation. Future studies may benefit from this type of framing given the close ties between public policy and governmental public health agencies.

De-implementation of ineffective programs in public health departments is an emerging field. To date, research on de-implementation has been primarily focused on the clinical healthcare setting (5). A notable exception is a work by McKay et al., who developed a conceptual narrative of de-implementation in public health by reviewing research and frameworks developed on de-implementation in the public health and healthcare settings. The authors discuss how once an intervention is identified as needing to be de-implemented, there are several socio-political factors that will affect if the intervention is actually de-implemented, including individual attitudes, political will, stakeholder belief that an intervention should be adopted, beliefs that there is an alternative intervention available, and characteristics of the interventions themselves (5). Many of the strategies for ending ineffective programs identified by practitioners in this study relate to addressing these socio-political factors in the de-implementation process, for example, maintaining positive relationships with stakeholders and communicating meaningfully with decision-makers. Though the research on de-implementation in public health is lacking, there are several studies that have examined factors related to the themes identified in this analysis.

Evaluation has been found by other researchers to be an important facilitator of ending ineffective programs. In a survey of 376 local health department practitioners by Allen et al., lack of program evaluation was cited by 28% of participants as a top reason for why programs continued that should have ended (27). As the purpose of the evaluation in public health is to determine the effectiveness of a given intervention and/or assess and improve the quality of the intervention it, makes sense that evaluation plays an important role in identifying and building a case to de-implement an ineffective program (28). Gathering and communicating research evidence and local data are key components in the resource allocation framework by Harris et al. (9).

Other researchers have made a case for why an intervention should be saved where possible. A longitudinal study by Pinto et al. examined 379 HIV providers' experiences in New York City when the Centers for Disease Control and Prevention began to de-implement HIV prevention interventions that had been in place for 20 years in favor of more effective approaches that had better supporting evidence. The study found that the de-implementation of these interventions had some unintended negative consequences, including downsizing of staff due to decreased funding and a reduction in technical resources and assistance that had come from the intervention before it ended (29–31). A commentary on de-implementation by Norton and Chambers also discusses at length potential unintended negative consequences, including downsizing, loss of trust by intervention recipients, and loss of revenue, that can result from de-implementing low-value health interventions (22). This study and commentary support the advice our participants gave to examine if a program needs to end before ending it because it may cause unintended ripple effects on the agency. Additionally, a study by Adam et al. (32) developed a framework to examine characteristics related to the continuation or termination of public organizations. This framework can be adapted to examine de-implementation of programs and identify de-implementation strategies. The framework suggests that adaptations are a necessary strategy for making ineffective programs effective, which may prevent the need for de-implementation. However, the authors warn that selecting and implementing the appropriate adaptations can be very difficult. This research also seems to support our participants' advice that adapting a program can be an important tool for making previously ineffective programs effective thus preventing the need for de-implementation.

Transparently communicating about program adjustments has been highlighted in other work about de-implementation of ineffective programs. Niven et al. conducted a review of articles describing the process of de-implementation, predominately in the clinical healthcare sector. The researchers developed a framework based on 109 studies on de-implementation (6). At the center of the framework is stakeholder engagement which indicates that it takes place at all stages of de-implementation from assessing interventions to see if they should be de-implemented to assessing barriers and facilitators to de-implantation, and sustaining the de-implementation. This framework is in line with the advice from our participants. It highlights the importance of communicating with stakeholders early in the process of de-implementation and the importance of listening to stakeholders and getting their feedback.

The importance of partnerships in the de-implementation of ineffective programs was also found in other studies. Pinto et al., discussed above, study found that de-implementation had negative impacts on interprofessional collaboration and partnerships as HIV service providers became more reluctant to send referrals to other agencies that do not engage in mutual referral-making (29–31). The ending of these HIV interventions ceased the partnerships that these programs had created, which may have affected the providers' ability to coordinate patient care. Additionally, Norton and Chambers discuss that stakeholder buy-in is an important strategy for effective de-implementation in healthcare settings (22). This study and commentary support what our participants advised, that one must be very considerate in the communication with and treatment of partners when an ineffective program needs to be de-implemented. This care in communicating with partners is important not only for effectively ending the program but also for persevering relationships with partners for the future.

Participants in this study cited the need for using communication strategies that resonate with decision-makers as an important factor in de-implementation of ineffective programs. The organizational behavior literature provides further insights into what types of communication are effective for an employee having their request granted by a superior. A study by Garner et al. examined what types of supervisor-employee communication interactions are most likely to result in the employee “getting what they want out of the conversation” (33, 34). Significant correlations with conversational effectiveness, in order of effectiveness, included solution presentation, repetition, circumvention, ingratiation, exchange, inspiration, and direct factual appeals. However, more work needs to be done to understand how those working in public health can best communicate the need for de-implementation of an ineffective program to their unique stakeholders.

### Limitations

There are several limitations to this study including a limited sample size, participant self-censure, and participant self-report of ineffective programs and effective strategies for ending ineffective programs. As discussed in the methods, we selected states to invite participants from to be representative of various factors, including representation from each region of the US. It should be noted, however, that this study has limited generalizability, since it only includes representation from eight states. Additionally, due to the political nature of public health and its funding, participants sometimes self-censured their responses. Despite following IRB protocols and informing participants that their responses, state, and name would remain confidential, participants sometimes let us know they were leaving information out or asked us to redact pieces of information. Lastly, we relied on participants to self-report and define what makes an ineffective program and the effectiveness of the advice that they provided to end ineffective programs. We did not assess effectiveness or strategies objectively.

### Implications for Practice and Future Directions

To our knowledge, this is the first analysis to examine advice from public health practitioners on how to de-implement ineffective programs in public health departments. Understanding how public health departments effectively de-implement ineffective interventions is vital to make the best use of limited resources in public health. Many of the study findings are in line with the general evidence-based decision-making principles (12). These findings have several implications for public health practice. Firstly, public health interventions should have an initial evaluation plan and public health departments should build capacity for evaluation where lacking. Secondly, where possible, ineffective interventions should be adapted to become effective instead of ended in order to avoid unintended negative consequences of de-implementation. Thirdly, public health practitioners should be in frequent communication about their programs with decision-makers and stakeholders. Fourthly, public health practitioners should be sensitive to partners when de-implementing ineffective programs. Fifthly, public health practitioners need to communicate in ways that make sense and resonate with those that have decision-making power over programs ending. Adopting these practices and building capacity for evidence-based decision-making may help public health departments to identify and de-implement a program much earlier in the program life cycle.

More research is needed on factors affecting successful de-implementation processes of ineffective programs in the public health setting. Additionally, this paper examined de-implementation in the state health department setting, but future research is needed on local health departments, which may reveal different effective strategies for de-implementing ineffective programs in this setting.

## Conclusion

This study adds to the limited body of research on de-implementation in public health agencies. This analysis provides insight into how experienced SHD practitioners recommend ending ineffective programs which may be useful for others working at public health agencies. It also provides a guiding point for future researchers to systematically assess these strategies to further the understanding of de-implementation and its effect on public health programming.

## Data Availability Statement

The raw data supporting the conclusions of this article will be made available by the authors, without undue reservation.

## Ethics Statement

The studies involving human participants were reviewed and approved by Washington University in St. Louis Institutional Review Board (IRB# 201812062). Written informed consent for participation was not required for this study in accordance with the National Legislation and the institutional requirements.

## Author Contributions

ERW conducted interviews, coded interview transcripts, analyzed and interpreted interview themes, and led and contributed to writing the manuscript. PA and SM guided study design and interview guide development, coded interview transcripts, assisted in theme synthesis, and was a contributor in writing the manuscript. LF assisted in theme synthesis and was a contributor in writing the manuscript. MP managed the development of the interview guide, conducted interviews, coded interview transcripts, analyzed and interpreted interview themes, and was a contributor in writing the manuscript. SM-R guided study design and interview guide development, conducted interviews, coded interview transcripts, analyzed and interpreted interview themes, and led and contributed to writing the manuscript. RB guided study design and interview guide development and contributed to writing the manuscript. All authors read and approved the final manuscript.

## Author Disclaimer

The findings and conclusions in this paper are those of the authors and do not necessarily represent the official positions of the National Institutes of Health or the Centers for Disease Control and Prevention.

## Conflict of Interest

The authors declare that the research was conducted in the absence of any commercial or financial relationships that could be construed as a potential conflict of interest.

## Publisher's Note

All claims expressed in this article are solely those of the authors and do not necessarily represent those of their affiliated organizations, or those of the publisher, the editors and the reviewers. Any product that may be evaluated in this article, or claim that may be made by its manufacturer, is not guaranteed or endorsed by the publisher.
